# Differential Regulation of NF-*κ*B and Nrf2 by Bojungikki-Tang Is Associated with Suppressing Lung Inflammation

**DOI:** 10.1155/2018/5059469

**Published:** 2018-01-30

**Authors:** Soo Ryun Park, Kyun Ha Kim, Min Jung Kwun, Ji Yeon Lee, Ran Won, Chang Woo Han, Jun Yong Choi, Myungsoo Joo

**Affiliations:** ^1^School of Korean Medicine, Pusan National University, Yangsan 50612, Republic of Korea; ^2^Department of Biomedical Laboratory Science, Division of Health Sciences, Dongseo University, Busan 47011, Republic of Korea; ^3^Department of Internal Medicine, Korean Medicine Hospital, Pusan National University, Yangsan 50612, Republic of Korea

## Abstract

Bojungikki-tang (BT), an Asian herbal remedy, has been prescribed to increase the vitality of debilitated patients. Since a compromised, weakened vitality often leads to illness, BT has been widely used to treat various diseases. However, little is known about the mechanism by which BT exerts its effect. Given that BT ameliorates inflammatory pulmonary diseases including acute lung injury (ALI), we investigated whether BT regulates the function of key inflammatory factors such as NF-*κ*B and Nrf2, contributing to suppressing inflammation. Results show that BT interrupted the nuclear localization of NF-*κ*B and suppressed the expression of the NF-*κ*B-dependent genes in RAW 264.7 cells. In similar experiments, BT induced the nuclear localization of Nrf2 and the expression of the Nrf2-dependent genes. In a lipopolysaccharide-induced ALI mouse model, a single intratracheal administration of BT to mouse lungs ameliorated alveolar structure and suppressed the expression of proinflammatory cytokine genes and neutrophil infiltration to mouse lungs. Therefore, our findings suggest that suppression of NF-*κ*B and activation of Nrf2, by which BT suppresses inflammation, are ways for BT to exert its effect.

## 1. Introduction

The acute lung injury (ALI) or acute respiratory distress syndrome (ARDS) is an inflammatory lung disease that occurs in critically ill patients with 30 to 40% of mortality rate [1]. The mortality is disproportionately higher among elderly patients with ARDS [2, 3], suggesting that aging affects the outcome of ARDS. Etiology of ALI/ARDS includes trauma, sepsis, bacterial and viral infection, and other immunologic challenges [1, 4]. Although pulmonary inflammation is an essential tool wielded to fend off those challenges, dysregulated inflammation in the alveoli leads to ALI/ARDS. Therefore, taming lung inflammation has been a therapeutic target for ALI/ARDS.

Alveolar macrophages, residential cells in the lung, play a pivotal role in regulating inflammation [5–7]. When a bacterial substance such as lipopolysaccharide (LPS) binds to its cognate receptor, Toll-like receptor 4 (TLR4), on macrophages, the signaling cascade occurs, resulting in NF-*κ*B activation [8]. Activation of NF-*κ*B contributes to the expression of cytokines such as TNF-*α*, IL-1*β*, IL-6, and IL-8 [8], which collectively promote inflammation. Another cell type prominently detected in the milieu of acute inflammation is neutrophils [9]. Accumulation of neutrophils in the alveolar space is accompanied by pulmonary edema and alveolar-capillary barrier damage; the level of neutrophils in the bronchoalveolar lavage (BAL) fluid is generally correlated to the severity and bad outcome in patients [10]. However, targeted drugs annihilating those cytokines have failed to control ALI/ARDS [11]. The ineffectiveness of these drugs may stem from the fact that ALI/ARDS involves a complex array of factors including monocytes, platelets, coagulation factors, reactive oxygen, and nitrogen species [12].

Cumulative evidence suggests that Nrf2 plays a protective role against oxidative damage and inflammation [13, 14]. Nrf2 is a basic leucine zipper transcriptional factor kept in the cytoplasm by Keap1 in normal conditions. Keap1, an actin-anchored protein, binds to and mediates ubiquitination of Nrf2, which leads to the ubiquitin-dependent degradation of Nrf2 by the 26S proteasome in the cytoplasm. Degradation of Nrf2 drives the level of Nrf2 to be low in the nucleus, resulting in a minimal and low transcriptional activity of Nrf2. However, in an inflammatory or oxidative milieu, Keap1 stops promoting the ubiquitination of Nrf2, letting Nrf2 be accumulated in the nucleus [13, 14]. Accumulated in the nucleus, Nrf2 induces the expression of Nrf2-regulated genes, such as NAD(P)H:quinone oxidoreductase (NQO1), heme oxygenase-1 (HO-1), and glutamate-cysteine ligase catalytic (GCLC) subunit, the role of which guards host cells against the oxidative stress [13, 15]. The critical role of Nrf2 in suppressing inflammation is demonstrated in a series of studies with Nrf2-knockout (KO) mice. For instance, Nrf2 KO mice were found to be more susceptible to developing lupus-like autoimmune nephritis [16], a greater lung inflammation in response to LPS and TNF-*α* [14], and a prolonged neutrophil infiltration into the inflammatory sites [17]. Given the importance of NF-*κ*B and Nrf2 in regulating inflammation and the fact that strategies that block a single pathway have been ineffective [12], development of drugs targeting Nrf2, along with NF-*κ*B, has been proposed [18].

Bojungikki-tang (BT), which is known as Bu Zhong Yi Qi Tang or Hochu-ekki-to in Chinese and Japanese medicine, respectively, is one of the old and widely used herbal formulas in East Asia [19, 20]. Traditionally, BT has been prescribed for feeble patients or the elderly to enhance vitality and to help them regain a physiological balance and thus a healthy condition [21, 22]. Therefore, BT has been used to treat a broad spectrum of diseases, including inflammatory diseases such as allergic rhinitis, upper respiratory infection, chronic bronchitis, chronic hepatitis, chronic nephritis, and corneal ulcer [19, 20, 23]. Concordantly, BT has shown anti-inflammatory effects in human [22–24] and in mice [25]. A recent study identifies the major constituents of BT, which include astragaloside IV, calycosin, glycyrrhizic acid, ferulic acid, hesperidin, and diclofenac [26]. Given that they have been reported to regulate Nrf2 activity [27–33], the anti-inflammatory function of BT is likely mediated by Nrf2. However, in light of the pivotal role of NF-*κ*B and Nrf2 in regulating inflammation, whether the anti-inflammation effect of BT involves NF-*κ*B and Nrf2 is worth investigating. Here, we show that BT differentially regulated these two factors, resulting in suppressed lung inflammation in an LPS-triggered ALI mouse model. Our results suggest that downregulating excessive inflammation is part of BT function.

## 2. Materials and Methods

### 2.1. Plant Materials

Bojungikki-tang (BT) or Hochu-ekki-to (TJ-41) was obtained from Tsumura Co., Ltd. (Tokyo, Japan). BT (TJ-41) contains a mixture of spray-dried hot water extracts of Astragali radix (16.7%), Atractylodis lanceae rhizoma (16.7%), Ginseng radix (16.7%), Angelicae radix (12.5%), Bupleuri radix (8.3%), Zizyphi fructus (8.3%), Aurantii nobilis pericarpium (8.3%), Glycyrrhizae radix (6.3%), Cimicifugae rhizoma (4.2%), and Zingiberis rhizoma (2.0%). BT was dissolved in PBS and filter-sterilized prior to the experiment.

### 2.2. Reagents and Antibodies

TLR4-specific LPS (*Escherichia coli* O55:B5) was purchased from Alexis Biochemical (San Diego, CA, USA). Sulforaphane and anti-HA antibody (H6908) were obtained from Sigma-Aldrich (St. Louis, MO, USA). Antibodies against p65 (C-20), Nrf2 (H-300), lamin B (C-20), and lamin A/C (H-110) were from Santa Cruz Biotechnology (Santa Cruz, CA, USA).

### 2.3. Cell Culture

RAW 264.7 cells were obtained from American Type Culture Collection (ATCC, Rockville, MD, USA) and cultured in Dulbecco's Modified Eagle's Medium (DMEM) supplemented with 10% (v/v) heat-inactivated FBS (Thermo Scientific, MA, USA), 100 U/ml penicillin (Thermo Scientific), and 100 *μ*g/ml streptomycin (Thermo Scientific), at 37°C under 5% CO_2_ in humidified culture chamber.

### 2.4. Assessment of Cytotoxicity

Cytotoxicity caused by BT was assessed with an MTT-based colorimetric assay and the protocol provided by the manufacturer (Sigma-Aldrich). Ten thousand RAW 264.7 cells were seeded in 24-well culture plates and treated with various concentrations of BT for 16 h. The optical density (OD) of formazan was measured at 550 nm with a microplate reader.

### 2.5. Measurement of Reactive Oxygen Species (ROS)

ROS were measured by flow cytometry. Briefly, after BT treatment, 1 × 10^6^ RAW 264.7 cells were treated with 10 *μ*M chloromethyl- (CM-) H_2_DCFDA, incubated at 37°C for 30 min, and then washed with PBS. Fluorescence was detected by BD FACS Canto II flow cytometer (BD Biosciences, San Jose, CA, USA) at the excitation wavelength of 488 nm and the emission wavelength of 525 nm.

### 2.6. Total RNA Extraction and Semiquantitative RT-PCR

Total RNA was isolated from cells and right lung using TRIZOL (GeneAll, Korea) per the manufacturer's protocol. 2 *μ*g of total RNA was reverse-transcribed by M-MLV reverse transcriptase (Promega, Madison, WI, USA). The resultant cDNA was amplified with* Taq* PCR × DNA polymerase (Thermo Scientific) and a set of specific primers: the primers for NQO-1 were 5′-GCAGTGCTTTCCATCACCAC-3′ and 5′-TGGAGTGTGCCCAATGCTAT3′; the primers for HO-1 were 5′-TGAAGGAGGCCACCAAGGAGG-3′ and 5′-AGAGGTCACCCAGGTAGCGGG-3′; the primers for GCLC were 5′-CACTGCCAGAACACAGACCC-3′ and 5′-ATGGTCTGGCTGAGAAGCCT-3′; the primers for IL-1*β* were 5′-GTGTCTTTCCCGTGGACCTT3′ and 5′-TCGTTGCTTGGTTCTCCTTG-3′; the primers for TNF-*α* were 5′-CTACTCCTCAGAGCCCCCAG-3′ and 5′-AGGCAACCTGACCACTCTCC-3′; and the primers for GAPDH were 5′-GGAGCCAAAAGGGTCATCAT-3′ and 5′-GTGATGGCATGGACTGTGGT-3′. PCR started at 95°C for 5 min and subsequent 20 to 30 cycles of denaturation for 30 sec at 95°C, annealing for 30 sec at 58°C, and extension for 40 sec at 72°C with a final extension for 7 min at 72°C. PCR products were separated in 1.2% agarose gels in 1x TBE buffer at 100 V for 30 min, stained with SYBR safe DNA gel stain (Thermo Scientific), and visualized under LED light. The level of GAPDH cDNA from each sample was used to normalize PCR efficiency among different samples. GAPDH was also used as internal control to evaluate relative expressions of target genes. Relative expression of each gene over GAPDH was determined by densitometer software ImageJ (NIH, Bethesda, MD, USA).

### 2.7. Western Blot Analysis of Nuclear Proteins

Nuclear proteins were extracted from RAW 264.7 cells (5 × 10^6^ cells) using NE-PER nuclear extraction kit and the manufacturer's protocol (Thermo Scientific). The amounts of proteins were measured by Bradford assay (Bio-Rad, Hercules, CA, USA). Equal amounts of proteins were fractionated by SDS-PAGE and then transferred to PVDF membrane (Bio-Rad). Blots were blocked for at least 1 h with 5% nonfat dry milk prior to incubation with appropriate antibodies at 4°C overnight. After incubation with secondary antibodies conjugated with HRP for 1 h at room temperature, specific bands of interest were revealed by chemiluminescence (SuperSignal® West Femto, Thermo Scientific).

### 2.8. Animals

Experimental procedures for mouse experiment were approved by the Institutional Animal Care and Use Committee of Pusan National University (approval number: PNU-2013-0322). C57BL/6 mice (Jackson Laboratory, Bar Harbor, ME, USA) aged 7 to 10 weeks received a single intratracheal (i.t.) LPS (2 mg/kg body weight). LPS was loaded in a MicroSprayer® Aerosolizer (Model IA-1C, Penn-Century, Inc., USA) and delivered to the lung via the intratracheal passage. At 2 h after LPS treatment, 1 or 10 mg/kg body weight of BT in 25 *μ*l of PBS was loaded in the MicroSprayer® Aerosolizer and delivered to the lung in a similar way. At 16 h after LPS treatment, bronchoalveolar lavage (BAL) was performed through trachea that was open through a midline incision and cannulated with a sterile 24-gauge intravascular catheter. Lungs were lavaged two times using 1 ml of sterile PBS. Inflammatory cells in BAL fluid were counted as described elsewhere [34]. Three hundred cells in total were counted, and one hundred cells in each microscopic field were scored. The mean number of cells per field was reported. For the analysis of lung tissue, the whole lung was perfused with PBS and then inflated with fixatives. Lungs embedded in paraffin were cut in 5 *μ*m thickness and stained with a hematoxylin and eosin (HE) staining method. Three separate HE-stained sections were evaluated in 100x microscopic magnifications per mouse.

### 2.9. Measurement of Myeloperoxidase Activity

Myeloperoxidase (MPO) activity in lung homogenates of mice was measured using the myeloperoxidase fluorometric detection kit and the manufacturer's instruction (Enzo Life Sciences International, Inc., New York, USA). Data were presented as unit/g tissue.

### 2.10. Statistical Analysis

To compare the results among groups, one-way analysis of variance (ANOVA) tests with Tukey's post hoc test were used (InStat, GraphPad Software, Inc., San Diego, CA). *P* values < 0.05 were considered significant. All experiments were performed at least three times independently to ensure the reproducibility of results.

## 3. Results

### 3.1. Suppression of NF-*κ*B Activity by Bojungikki-Tang

**Figure 1:**
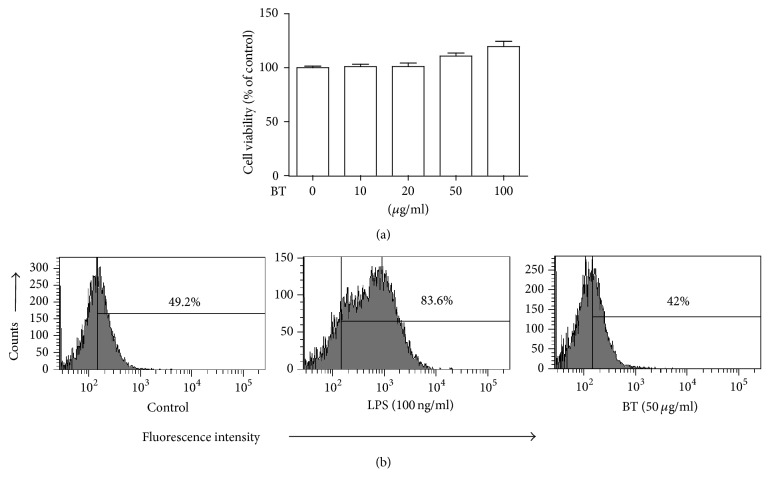
Cytotoxic effect of BT. (a) RAW 264.7 cells were treated with varying amounts of BT for 16 h. The cytotoxicity induced by BT was measured by MTT assay. Data are shown as the mean ± SEM of three independent measurements. There was no statistical difference among different groups. (b) RAW 264.7 cells were treated with LPS (100 ng/ml) or BT (50 *μ*g/ml) for 16 h. Cells producing intracellular ROS were determined by a flow cytometer. The percentile of ROS-positive cells is shown in the upper right side of each panel. All experiments were repeated three times independently and representative results are shown.

Prior to the experiment, we determined the range of the amounts of Bojungikki-tang (BT), within which no cytotoxicity is apparent. For this, RAW 264.7 cells were treated with varying amounts of BT (10 *μ*g/ml to 100 *μ*g/ml) for 16 h and the cytotoxicity inflicted by BT was measured by an MTT assay. As shown in Figure 1(a), BT did not exhibit any cytotoxicity within the range of 10 *μ*g/ml to 100 *μ*g/ml of BT. Since reactive oxygen species (ROS) produced during inflammatory reaction inflict harm on the host cells, we also tested whether BT induces ROS production. After treatment with CM-H_2_DCFDA, RAW 264.7 cells positive with fluorescent H_2_DCFDA, which was converted from CM-H_2_DCFDA by ROS, were scored by a flow cytometer. As shown in Figure 1(b), RAW 264.7 cells treated with BT exhibited fluorescence similar to controls (42% versus 49.2%), while a significant increase in fluorescence intensity was detected in the LPS-treated RAW 264.7 cells (83.6%). Since less than 50 *μ*g/ml of BT elicited neither cytotoxicity nor ROS production, we decided to test the anti-inflammatory effect of BT within the range of 1 to 20 *μ*g/ml of BT.

**Figure 2:**
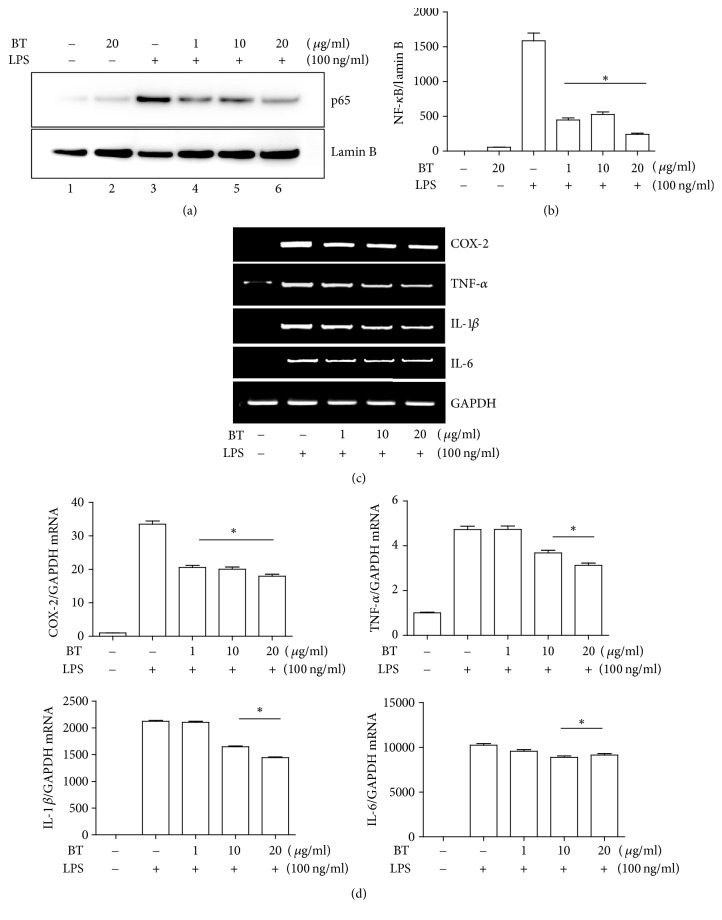
BT suppresses the transcriptional activity of NF-*κ*B. (a) RAW 264.7 cells, pretreated with PBS (lanes 1 and 2) or the indicated amounts of BT for 16 h (lanes 2 and 4 to 6), were stimulated with TLR4-specific LPS (100 ng/ml) for 30 min (lanes 3 to 6). Nuclear proteins were isolated from the cells and analyzed by immunoblotting of p65 RelA. For ensuring equal loading, the membrane was stripped and reprobed for lamin B. (b) The intensity of each band was measured by ImageJ and calculated over lamin B. Data represent the mean ± SEM of three independent measurements. ^*∗*^*P* was less than 0.05, compared to the control treated with LPS only. (c) RAW 264.7 cells were treated with BT for 16 h and subsequently with TLR4-specific LPS (100 ng/ml) for 4 h. The levels of COX-2, TNF-*α*, IL-1*β*, and IL-6 were analyzed by a semiquantitative RT-PCR. (d) The intensity of each PCR band was measured by ImageJ and calculated over that of a housekeeping gene GAPDH. Data represent the mean ± SEM of three independent measurements. ^*∗*^*P* was less than 0.05, compared to the control treated with LPS only.

Given the reports showing an anti-inflammatory activity of BT, we first examined whether BT suppresses the proinflammatory factor NF-*κ*B. RAW 264.7 cells were treated with varying amounts of BT (1 *μ*g/ml to 20 *μ*g/ml) for 16 h and then with LPS (100 ng/ml) for 30 min to activate NF-*κ*B. Since activated NF-*κ*B rapidly migrates to the nucleus, nuclear proteins were fractionated and analyzed by immunoblotting of p65 RelA, a subunit of NF-*κ*B [35]. As shown in Figure 2(a), LPS treatment induced the nuclear localization of p65 RelA (lane 3), which was decreased by increasing amounts of BT. A densitometric analysis indicated that BT significantly decreased the nuclear localization of p65 RelA (Figure 2(b)). To determine whether the nuclear NF-*κ*B diminished by BT is correlated with the decreased expression of NF-*κ*B-dependent genes, we treated RAW 264.7 cells with varying amounts of BT as in Figure 2(a) and then with LPS (100 ng·ml) for 4 h. Total RNA was extracted from the cells and analyzed by a semiquantitative RT-PCR for the expression of some of representative NF-*κ*B dependent genes, such as COX-2, TNF-*α*, IL-1, and IL-6. As shown in Figure 2(c), treatment of RAW 264.7 cells with BT also suppressed the expression of the NF-*κ*B-dependent genes. Densitometric analysis of these genes shows that the reduction was statistically significant (Figure 2(d)), suggesting that the nuclear NF-*κ*B which decreased by BT is associated with the suppressed expression of the NF-*κ*B-dependent genes. Albeit statistically significant, the degree of the expression of these gene suppressed by BT appeared not robust (Figure 2(d)), suggesting that while the anti-inflammatory function of BT involves the suppression of NF-*κ*B, another factor may also contribute to the anti-inflammatory effect of BT.

### 3.2. Activation of Nrf2 Activity by Bojungikki-Tang

**Figure 3:**
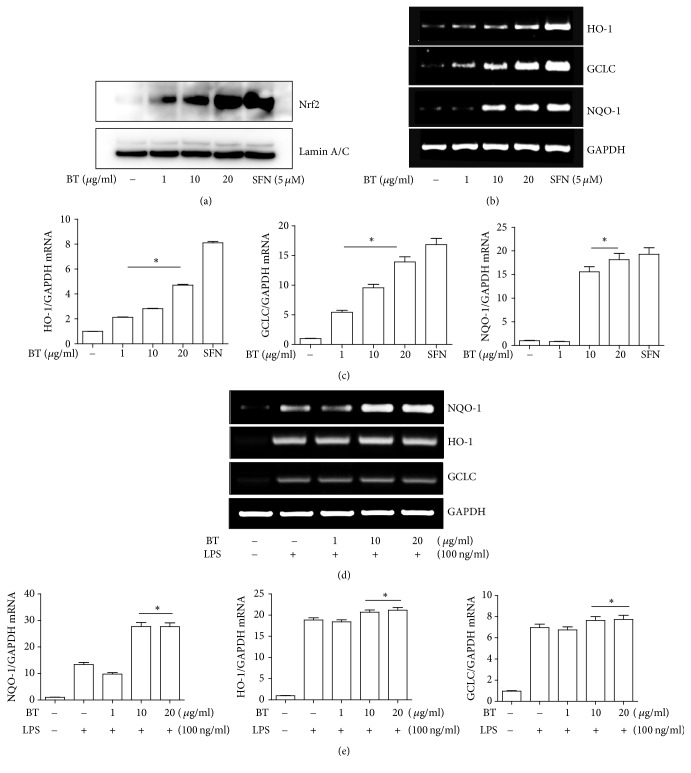
BT activates the transcriptional activity of Nrf2. (a) RAW 264.7 cells were treated with varying amounts of BT for 16 h. As for Nrf2 control, cells were similarly treated with 5 *μ*M of sulforaphane (SFN), a potent Nrf2 activator. Nuclear proteins were isolated from these cells and analyzed by immunoblotting of Nrf2. The membrane was stripped and blotted with *α*-lamin A/C antibody for nuclear protein controls. A similar experiment was repeated at least three times. (b) RAW 264.7 cells were treated similarly to (a), and total RNA was extracted and analyzed by a semiquantitative RT-PCR for the expression of HO-1, GCLC, and NQO-1. (c) PCR bands in (b) were analyzed by ImageJ, and relative expressions of individual genes were shown in graphs after being normalized to GAPDH. (d) RAW 264.7 cells were treated with BT for 16 h and subsequently with TLR4-specific LPS (100 ng/ml) for 4 h. A semiquantitative RT-PCR was performed for the expression of HO-1, GCLC, and NQO-1. Resultant PCR bands were analyzed by ImageJ, and relative expressions of individual genes were shown in graphs after being normalized to GAPDH (e). Data represent the mean ± SEM of three independent measurements. ^*∗*^*P* was less than 0.05, compared to untreated controls.

As Nrf2 is a critical factor in suppressing inflammation, we next investigated whether Nrf2 is involved in the anti-inflammatory function of BT. RAW 264.7 cells were treated with varying amounts of BT (1 *μ*g/ml to 20 *μ*g/ml) for 16 h. Since, when activated, Nrf2 moves to the nucleus, nuclear proteins were isolated from the variously treated cells and analyzed by immunoblotting of Nrf2 to reveal the nuclear Nrf2. As shown in Figure 3(a), BT treatment induced a robust nuclear localization of Nrf2, suggesting that BT activates Nrf2. To examine whether BT inducing the nuclear localization of Nrf2 is correlated with the expression of Nrf2-dependent genes, we isolated total RNA from RAW 264.7 cells, which were treated with BT similarly to Figure 3(a), and performed a semiquantitative RT-PCR for the mRNA of HO-1, GCLC, and NQO-1. As shown in Figure 3(b), BT treatment induced the expression of these genes. The densitometric analysis shows that the expression of these genes was significantly increased, compared to the untreated control (Figure 3(c)). Since LPS treatment produces ROS (Figure 1(b)) that activate Nrf2 [36], LPS alone robustly induced the expression of Nrf2-dependent genes (2nd lanes in Figure 3(d)). In RAW 264.7 cells treated with LPS, additional treatment with BT further increased the expression of those genes (4th and 5th lanes). However, the degree of Nrf2 activated by BT appears marginal albeit statistically significant (4th and 5th columns in Figure 3(e)). It is likely that most Nrf2 was activated by LPS; only a small population of Nrf2 became further activated by BT. Nevertheless, combined with the results in Figure 1(b), these findings indicate that BT activated Nrf2 without the mediation of ROS, suggesting that BT activating Nrf2 contributes to the anti-inflammatory function of BT.

### 3.3. Bojungikki-Tang Reduces Lung Inflammation in an LPS-Induced ALI Mouse Model

**Figure 4:**
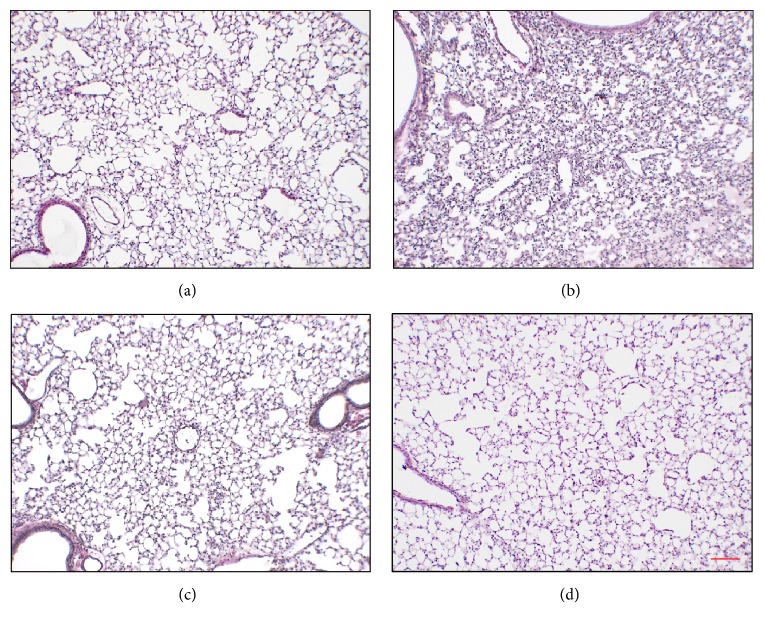
BT ameliorates alveoli structure of mouse lungs. C57BL/6 mice (*n* = 5 per group) received a single i.t. PBS (a) or 2 mg/kg of i.t. LPS ((b), (c), and (d)). Two hours later, mice received 1 mg/kg (c) or 10 mg/kg body weight of i.t. BT (d). Mouse lungs were harvested at 24 h after LPS treatment and stained with HE for histological examination (magnification, ×100, and bar = 100 *μ*m). Representatives of at least five different areas of a lung are shown.

Since BT suppressed NF-*κ*B and activated Nrf2, we examined whether BT suppresses inflammation. As inflammation involves various cell types in an organ, we used an LPS-induced ALI/ARDS mouse model to examine the anti-inflammatory effect of BT. Six-week-old C57BL/6 mice (*n* = 5/group) received an intratracheal (i.t.) administration of LPS (2 mg/kg body weight) or PBS and 2 h later two different doses of a single i.t. BT (1 mg/kg or 10 mg/kg body weight), which were determined after being converted from the two lowest amounts of BT (1 *μ*g/ml and 10 *μ*g/ml) that regulated the activity of NF-*κ*B and Nrf2 in RAW 264.7 cells. At 24 h after LPS treatment, bronchoalveolar lavage (BAL) was carried out to obtain inflammatory cells in the lung. Mouse lungs were harvested and HE-stained for the analysis of the alveolar structure. HE-stained lung sections (Figure 4) show that while delivery of PBS to mouse lungs did not induce changes in pulmonary structure (a), similar treatment with LPS induced a lung structure characteristic to inflammation (b). However, a single, low amount of i.t. BT (1 mg/kg body weight) seems to be sufficient to decrease the lung inflammation and a higher amount of i.t. BT (10 mg/kg body weight) appears to be more effective (d).

**Figure 5:**
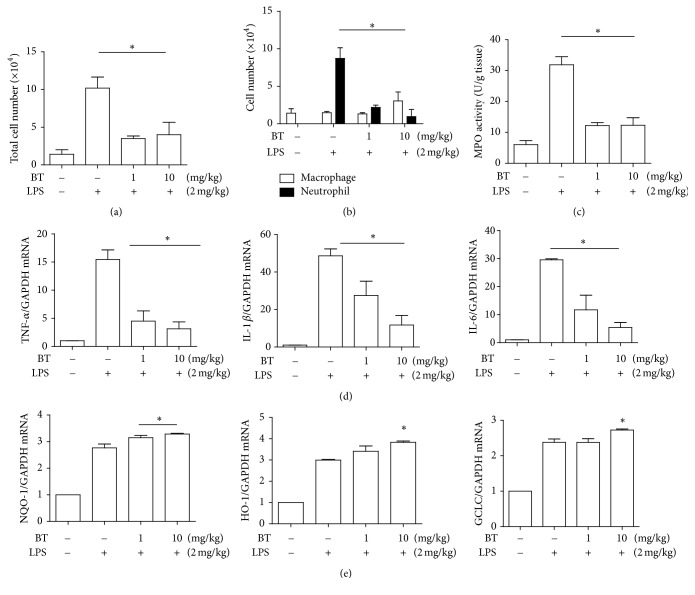
BT suppresses neutrophil infiltration to mouse lungs. C57BL/6 mice (*n* = 5 per group) received 2 mg/kg of i.t. LPS and then 2 h later two different amounts of i.t. BT were administered (1 mg/kg or 10 mg/kg body weight). Bronchoalveolar lavage (BAL) was conducted 24 h after LPS treatment. Total cells (a) and macrophages and neutrophils (b) in the BAL fluid were counted. Data are shown in the mean ± SEM of three independent measurements. ^*∗*^*P* was less than 0.05, compared to the LPS-treated mice (post-ANOVA comparison with Tukey's post hoc test). (c) The lysate was prepared from the lung and MPO assay was performed. Values are expressed as the mean ± SEM of 5 mice. ^*∗*^*P* was less than 0.05, compared to the LPS-treated mice (post-ANOVA comparison with Tukey's post hoc test). Total RNA was extracted from the lung tissue (*n* = 5 per group) and analyzed by a semiquantitative RT-PCR. PCR products were analyzed by ImageJ and normalized over GAPDH for the expressions of TNF-*α*, IL-1*β*, and IL-6 (d) and of Nrf2-dependent genes, such as NQO-1, HO-1, and GCLC (e). Representatives of three mice in each group are shown. ^*∗*^*P* was less than 0.05, compared to the LPS-treated mice (post-ANOVA comparison with Tukey's post hoc test).

To confirm the results, we performed differential counting of the cells in the BAL fluid. As shown in Figure 5(a), an i.t. LPS increased the number of cells infiltrated to the lung (2nd column), which was significantly diminished by an i.t. BT administration (3rd and 4th columns). Differential counting of the cells in BAL fluid (Figure 5(b)) shows that the majority of infiltrated cells were neutrophils (2nd closed column). While not significantly affecting the number of macrophages, an i.t. BT administration substantially decreased the level of neutrophils (3rd and 4th closed columns), suggesting that BT suppresses neutrophilic inflammation in the lung. To verify the result, we prepared the lysate from the lung tissue and measured myeloperoxidase (MPO) activity in it, as MPO is uniquely found in neutrophils [37]. As shown in Figure 5(c), an i.t. LPS elicited MPO activity (2nd column), which was significantly suppressed by two different amounts of an i.t. BT (3rd and 4th columns). These results are consistent with the finding that an i.t. BT decreased the neutrophil numbers in BAL as shown in Figure 5(b).

To confirm BT suppressing neutrophilic lung inflammation, we determined whether BT suppresses the expression of cytokines that promote inflammation. Total RNA was isolated from the lung tissue of mice treated as in Figure 4 (*n* = 5/group), which was analyzed by a semiquantitative RT-PCR for representative proinflammatory genes including IL-6, TNF-*α*, and IL-1*β*. As shown in Figure 5(d), an i.t. LPS induced the expression of these genes (2nd columns in each panel), which was significantly suppressed by two different amounts of an i.t. BT (3rd and 4th columns in each panel). To test whether the i.t. BT enhances the activity of Nrf2 in the lung, we also measured the expression of Nrf2-dependent genes in the lung in a similar fashion. Like Figures 3(d) and 3(e), an i.t. LPS induced the expression of Nrf2-dependent genes (2nd columns in Figure 5(e)), which was increased further by an i.t. BT in a statistically significant manner (2nd and 4th columns). Together, these results show that BT suppresses neutrophilic inflammation in mouse lungs.

## 4. Discussion

Unbalanced homeostasis or compromised vitality has been known to make people vulnerable to pathologic challenges, succumbing to diseases. Since BT has been used to help debilitated people regain their vigor and vitality, BT has been prescribed for patients to overcome various illnesses [20]. Consequently, the target diseases of BT have been unspecified and rather broad. However, recent studies have shown the effectiveness of BT in treating inflammatory diseases [23, 38]. BT was reported to be effective in treating a subset of chronic obstructive pulmonary diseases (COPD) [38] and rhinitis patients [23]. Notwithstanding these findings and a long history of clinical use of BT, the mechanism by which BT can exert its effect to suppress inflammation remains less understood. Since a recent study that identifies the major constituents of BT suggests that BT may suppress inflammation by modulating Nrf2 activity [26] and both NF-*κ*B and Nrf2 are key factors in regulating inflammatory response, we asked whether BT suppresses inflammation by regulating the activities of these two factors. Our results show that BT suppressed the transcriptional activity of a proinflammatory factor, NF-*κ*B, while activating the transcriptional activity of an anti-inflammatory factor, Nrf2. In an LPS-induced ALI/ARDS mouse model, an i.t. BT administration diminished neutrophilic lung inflammation, with increased Nrf2 activity. These results collectively suggest that BT is capable of suppressing acute inflammation, the effect of which is likely mediated by suppressing NF-*κ*B and activating Nrf2.

Our results implicated the suppression of NF-*κ*B in the anti-inflammatory function of BT. We show that BT significantly dampened the nuclear localization of NF-*κ*B and decreased the expression of the NF-*κ*B-dependent genes that promote inflammation. Therefore, it is likely that BT suppressing NF-*κ*B contributes to the anti-inflammatory function of BT. Our results also show that BT activated Nrf2, a critical anti-inflammatory transcription factor, and induced the expression of the Nrf2-dependent genes. BT activating Nrf2 is conceivable, given that the major compounds constituting BT activate Nrf2 [26]. However, it is not clear how these chemicals in BT collectively contribute to activation of Nrf2. It seems that BT did not induce the production of ROS, potent activator of Nrf2 in inflammatory milieu [39]. Therefore, it is likely that BT and its chemical constituents activated Nrf2 directly, not via ROS. Given the important roles of both factors in regulating inflammation, it is highly likely that both the suppression of NF-*κ*B and the activation of Nrf2 collectively contributed to BT suppressing inflammation.

Since inflammation is an orchestrated innate immune response that involves various immune cells, the effectiveness of BT in suppressing inflammation was tested in an LPS-induced ALI/ARDS mouse model. The doses of BT administered to mice were based on the doses used for cell experiment. Since 1 to 10 *μ*g/ml of BT regulated the activities of NF-*κ*B and Nrf2 in RAW 264.7 cells, we would like to test whether the equivalent amounts of BT, 1 to 10 mg/kg body weight, have a commensurable effect in suppressing inflammation in mice. It is notable that a single i.t. administration of 1 mg/kg body weight of BT was sufficient to suppress neutrophilic lung inflammation because 1 *μ*g/ml of BT, equivalent to 1 mg/kg body weight, minimally suppressed the expression of the NF-*κ*B-dependent genes and marginally induced the expression of the Nrf2-dependent genes in RAW 264.7 cells. The effective suppression of neutrophilic lung inflammation by BT can be explained by the fact that BT both suppressed NF-*κ*B and activated Nrf2 simultaneously, which was observed in RAW 264.7 cells. It is likely that because BT targeted both factors at the same time, the suppressive effect on inflammation could be manifested more effectively. Another factor that contributes to the high efficiency in suppressing inflammation could be the choice of delivery routes; unlike another study, we delivered BT to mouse lungs in an intratracheal rather than an oral route. Since BT was directly delivered to the lung, the loss of pharmaceutical effectiveness of BT was likely minimal, compared to oral administration in which the typical range of BT to mice is from 500 to 1,000 mg/kg body weight per day [40]. While it remains unknown whether the i.t. delivery of BT is clinically feasible, it is worthy to explore the i.t. route of administering herbal medicine and compare the effectiveness of the two different ways of administrating herbal medicine.

Although we used the i.t. delivery of BT, in principle, our results are consistent with the other report showing that BT prevents from lung inflammation in an LPS-induced ALI mouse model [41]. Since in our study BT was administered 2 h after the onset of lung inflammation, our results suggest a therapeutic rather than preventive effect of BT. Although we and other groups have studied the effect of BT on lung inflammation, it remains unclear how BT activates Nrf2, suppressing inflammation in the lung. A study has determined the tissue distribution of key constituents of BT in the rat, showing that substantial amounts of glycyrrhizic acid, saikosaponin D, and hesperidin are detectable in the lung [26]. Given that some of these chemicals regulate the activity of Nrf2 [31–33], it is conceivable that these constituents of BT migrated to and activated Nrf2 in the lung, contributing to suppressing inflammation. This study may bestow a relevance of i.t. delivery of BT to the lung.

## 5. Conclusion

Our results show that BT suppressed neutrophilic lung inflammation, along with suppression of NF-*κ*B and activation of Nrf2, suggesting that BT suppressing inflammation is associated with differential regulation of NF-*κ*B and Nrf2 activities. Although it is unclear how BT contributes to regaining vitality and helps recover from a tipped balance in patients, our results can serve as experimental evidence that BT suppressing inflammation is a way for BT to execute its effects.
